# Circadian Biology in Parasites: Beyond Known Mechanisms

**DOI:** 10.1146/annurev-micro-060424-051248

**Published:** 2025-10

**Authors:** Mukhtar Sadykov, Filipa Rijo-Ferreira

**Affiliations:** 1 Berkeley Public Health, Molecular and Cell Biology Department, University of California, Berkeley, California, USA; 2 Chan Zuckerberg Biohub San Francisco, San Francisco, California, USA

**Keywords:** infectious diseases, circadian clock, molecular parasitology, transcriptional regulation, posttranscriptional regulation, malaria

## Abstract

Circadian rhythms play a fundamental role in regulating biological processes across the tree of life. While these 24-h cycles are well-characterized in model organisms, their role in parasitic organisms has remained largely unexplored until recently. Here, we review emerging evidence that parasites possess intrinsic timekeeping abilities, focusing particularly on the malaria parasite *Plasmodium*. We examine two principal paradigms of biological timing: transcriptional-translational feedback loops and posttranscriptional feedback loops. Despite lacking canonical clock genes found in other eukaryotes, *Plasmodium* employs sophisticated regulatory machinery, including ApiAP2 transcription factors, chromatin regulators, and noncoding RNAs, that could form novel timing circuits. We discuss how these mechanisms might enable parasites to synchronize with host rhythms and optimize their development and transmission. Understanding these temporal regulatory networks could reveal new therapeutic strategies and expand our knowledge of biological timing mechanisms across evolution.

## 1. INTRODUCTION

**Figure 1:**
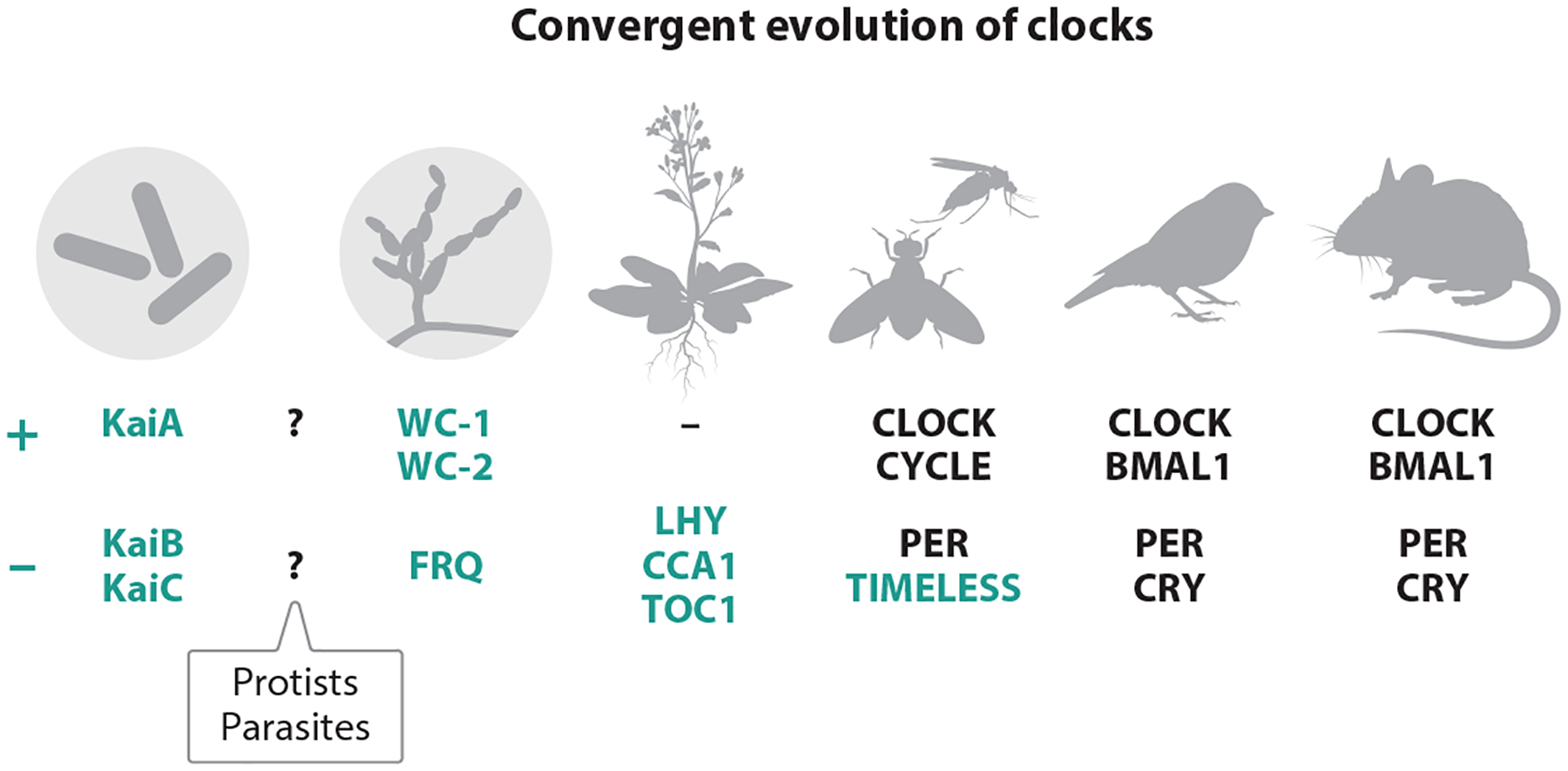
Conservation and diversity of circadian clock mechanisms across major taxonomic groups. The schematic presents organisms with known circadian clocks: mammals (human and mouse), birds, invertebrates (*Drosophila*, similar to *Anopheles* mosquitoes), plants (*Arabidopsis*), fungi (*Neurospora crassa*), and cyanobacteria (*Synechococcus elongatus*). Each main evolutionary group has evolved distinct molecular mechanisms to maintain circadian rhythms, demonstrating the convergent evolution of timekeeping systems. A summarized list of core activators (*plus sign*) and repressors (*minus sign*) of the clock mechanism is included. A question mark represents a lack of knowledge of the clock components or circuit of *Plasmodium* parasites' timekeeping mechanism. Abbreviations: CCA1, CIRCADIAN CLOCK ASSOCIATED 1; CRY, Cryptochrome; FRQ, Frequency; LHY, LATE ELONGATED HYPOCOTYL; PER, Period; TOC1, TIMING OF CAB EXPRESSION 1; WC, White Collar. Illustration design assisted by Takka Design.

Circadian rhythms are roughly 24-h physiological cycles, and are integral to regulating biological processes in organisms across the tree of life (8, 127). These rhythms orchestrate various functions, from gene expression to behavior, enabling organisms to anticipate and adapt to environmental changes such as light and temperature cycles (6). The molecular mechanisms underlying circadian rhythms have been extensively characterized in model organisms, revealing both conserved principles and lineage-specific innovations in timekeeping strategies (Figure 1) (33).

While circadian rhythms have been widely studied in mammals, plants, and other organisms, their role in parasitic organisms has remained largely unexplored until recently. New evidence suggests that these rhythmic cycles play a significant role in parasitic diseases, particularly in malaria parasites (122, 141). This discovery has expanded our understanding of parasite biology and raised important questions about how parasites synchronize their life cycles with those of their hosts and vectors to optimize replication and transmission (9, 98, 118).

The malaria parasite *Plasmodium* provides a compelling example of biological timing in parasites. Both the parasite's intraerythrocytic developmental cycle (IDC) and the fever patterns of infected hosts display multiple 24-h oscillations (77, 95, 148), suggesting that parasites possess internal clocks that synchronize with their hosts' circadian rhythms. These intrinsic rhythms persist even in arrhythmic host and culture conditions, indicating autonomous timing mechanisms (13, 122, 141). Such intrinsic rhythmic behaviors are not unique to malaria; similar cycles have been observed in another single-celled parasite—*Trypanosoma brucei* (123). Furthermore, parasitic worms such as *Schistosoma* and *Filaria* also undergo daily rhythms in their biology (49, 120), underscoring the potential universality of circadian regulation in parasitism.

Recent evidence that some of these rhythms are intrinsically regulated has opened new avenues for understanding parasite biology and its therapeutic implications. Disrupting these rhythms can significantly impact parasite fitness and transmission (105, 106), suggesting that targeting circadian mechanisms could provide novel treatment strategies (123, 125). However, the molecular components driving these rhythms remain largely unknown, as parasites lack canonical clock genes found in other eukaryotes.

This review synthesizes current knowledge of 24-h rhythms in malaria parasites and the molecular mechanisms underlying their temporal regulation. We examine two principal regulatory paradigms: transcriptional-translational feedback loops (TTFLs) and posttranscriptional feedback loops (PTFLs). Our analysis encompasses key molecular components, including transcription factors, chromatin regulators, and various DNA-binding proteins, that could participate in timekeeping. Finally, we discuss the role of noncoding RNAs (ncRNAs) in generating and maintaining these oscillations, providing a comprehensive framework for understanding the temporal architecture of parasitic infections across multiple mechanistic paradigms.

**Figure 2:**
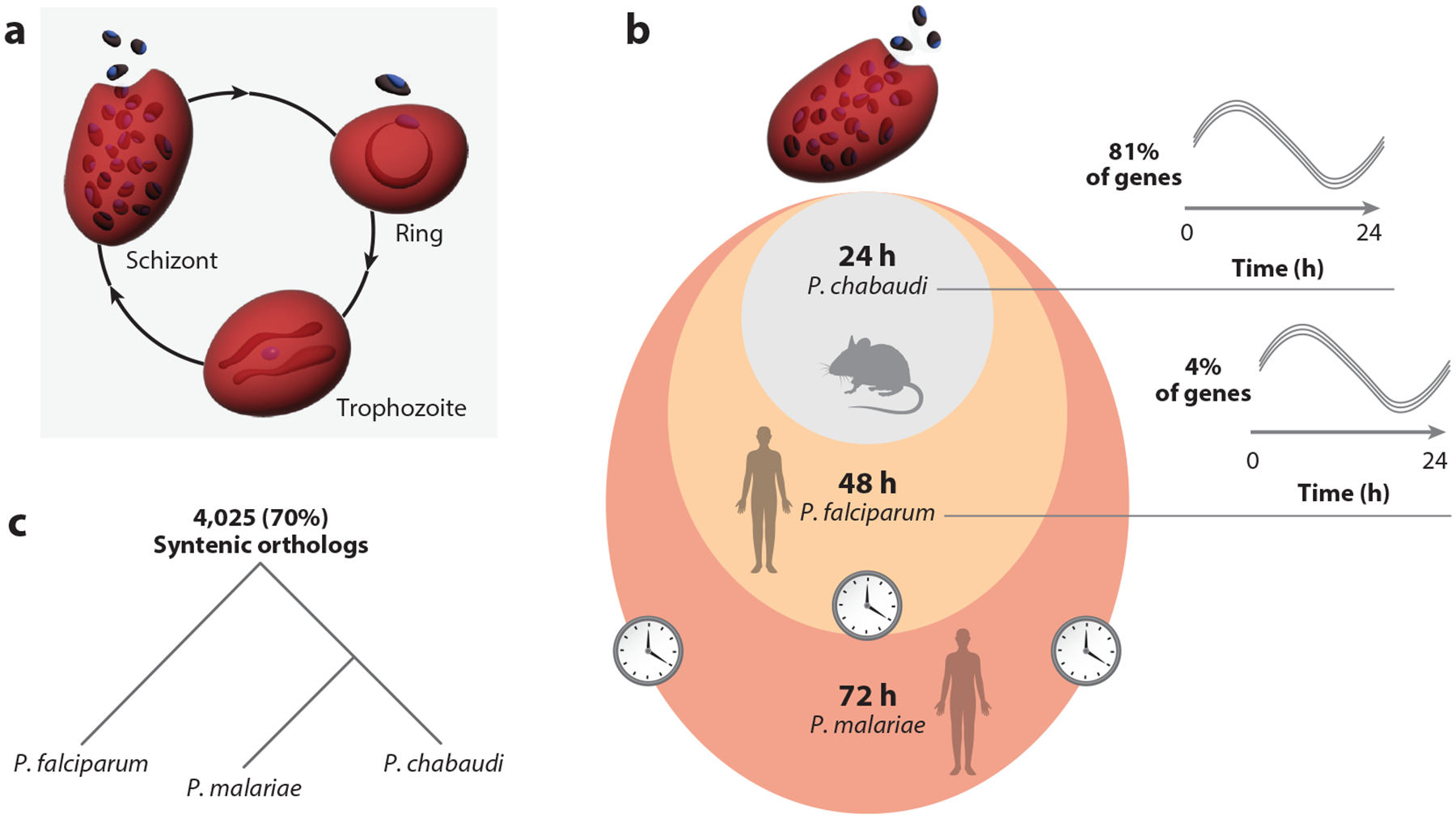
Developmental cycles and gene expression patterns across *Plasmodium* species. (*a*) The canonical replication cycle [intraerythrocytic developmental cycle (IDC)] shared by all *Plasmodium* species comprises three distinct morphological stages within the red blood cell: ring (early), trophozoite (middle), and schizont (late). During this cycle, parasites progress from initial invasion through growth and DNA replication to the final production of daughter merozoites. (*b*) Shown is a comparison of IDC duration and gene expression patterns across three *Plasmodium* species. The IDC length varies among species: *Plasmodium chabaudi* (24 h), *Plasmodium falciparum* (48 h), and *Plasmodium malariae* (72 h). *P. chabaudi* predominantly exhibits 24-h periodic gene expression (81% of genes) (122), while *P. falciparum* primarily shows 48-h periodicity (91% of genes) with a subset of 24-h periodic genes (4% of genes) (141). *P. malariae* displays the longest cycle at 72 h, though its transcriptional dynamics remain uncharacterized. (*c*) Despite their divergent cycle lengths, these *Plasmodium* species maintain high genetic conservation with 4,025 syntenic orthologs (70% of their genes) shared between them [using PlasmoDB ortholog analysis (3)]. This conservation suggests that fundamental timing mechanisms may have been preserved while the cycle duration evolved independently. Illustration design assisted by Takka Design.

What do we know about *Plasmodium* clocks, and what are the unknowns? Recent evidence suggests that malaria parasites possess intrinsic timekeeping abilities that allow them to synchronize their IDC with host circadian rhythms. Studies in rodent and human malaria models demonstrate that *Plasmodium* can maintain circadian rhythms in constant conditions, both in vivo (122) and in vitro (141), suggesting an endogenous clock mechanism (Figure 2). Similar rhythmicity has been observed in patients infected with *Plasmodium vivax*, showing direct coupling between host circadian rhythms and parasite IDCs (98). This conservation of timekeeping ability across *Plasmodium* species suggests it provides important fitness benefits, though the precise selective advantages remain to be fully understood.

While the existence of an intrinsic clock is becoming clear, the mechanisms by which malaria parasites sense and respond to host rhythms remain poorly understood. Several host cues have been proposed as potential zeitgebers (synchronizing cues), including melatonin (54), inflammatory cytokines and glucose metabolism (51), and isoleucine (118). However, direct evidence for any single factor being necessary or sufficient for entrainment is lacking. Initial studies also attempted to identify parasite components involved in maintaining rhythmicity, such as the serpentine receptor SR10 (143) and putative apicoplast transcriptional machinery (72), though their roles in timekeeping and whether they represent key regulatory elements remain to be determined.

It is also unclear how the parasite's intrinsic clock functions. While there is evidence of an intrinsic oscillator in *Plasmodium*, it lacks orthologs for typical circadian clock genes found in other eukaryotes, such as those involved in TTFLs, leaving fundamental questions about how its molecular clock is organized and regulated. This absence of canonical clock components is not surprising, as circadian systems have emerged through convergent evolution across different lineages (127). The molecular details of how these rhythms are generated and maintained remain unknown.

## 2. TRANSCRIPTIONAL-TRANSLATIONAL FEEDBACK LOOPS

**Figure 3:**
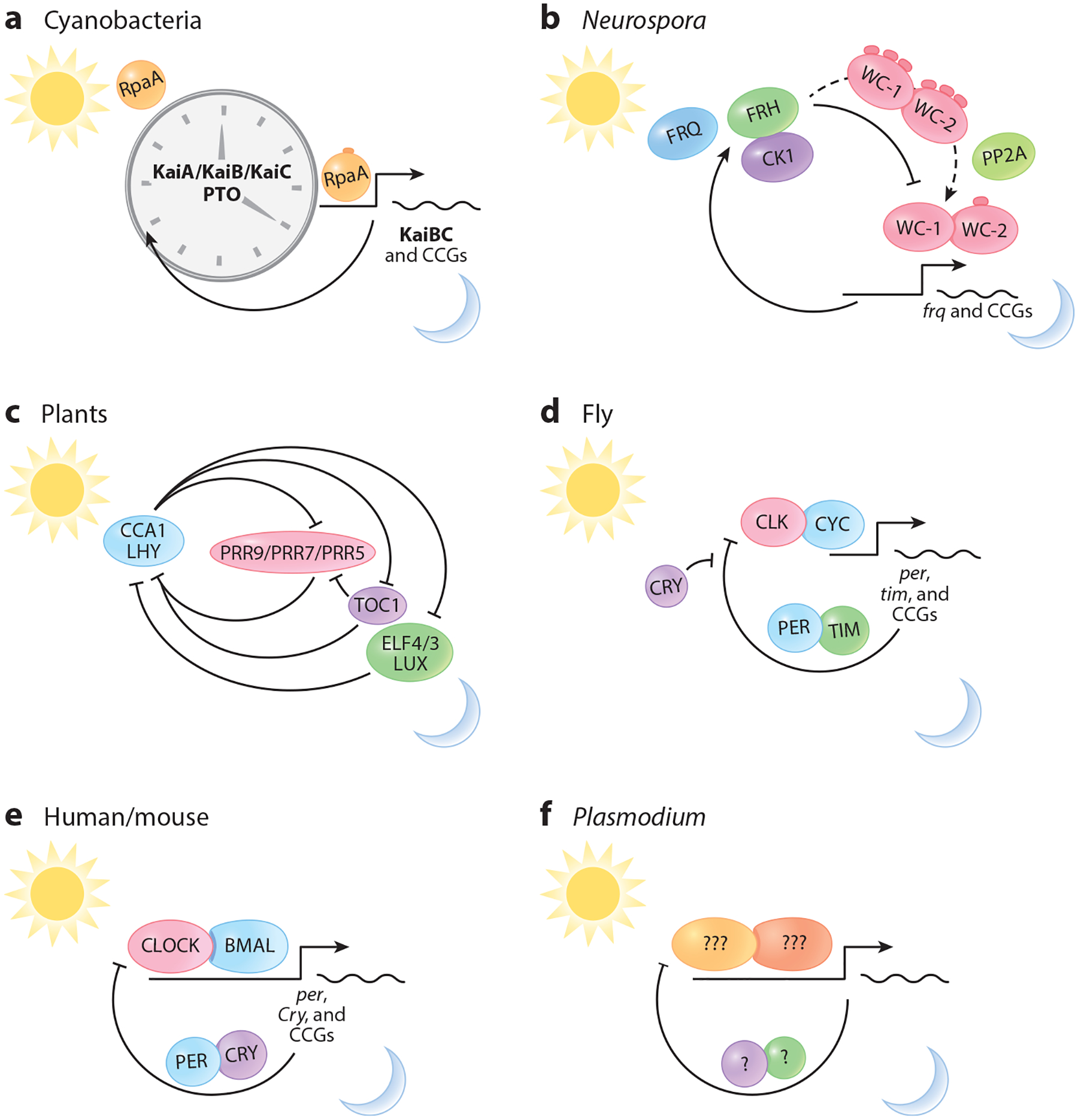
Molecular architecture of circadian clock systems across different kingdoms. Core molecular mechanisms of circadian clocks include (*a*) the KaiABC PTO in cyanobacteria, (*b*) FRQ/WCC-based TTFL in the fungus *Neurospora*, (*c*) CCA1/LHY and PRR-based reciprocal regulation in plants, (*d*) CLK/CYC-based TTFL in insects, such as the fly, (*e*) CLOCK/BMAL-based TTFL in mammals, such as humans and mice, and (*f*) unknown mechanisms in *Plasmodium*. Each system employs distinct molecular components while achieving similar 24-h rhythmicity. The percentage of cycling genes varies across organisms—mammals ~10–15% (169), invertebrates ~6% (57), plants 30–40% (26), fungi 10–40% (58), and cyanobacteria ~30% (61)—highlighting the extent of circadian regulation in different species. Abbreviations: CCA1, CIRCADIAN CLOCK ASSOCIATED 1; CCGs, clock-controlled genes; CK1, Casein Kinase 1; CLK, Clock; Cry, Cryptochrome; CYC, Cycle; ELF, EARLY FLOWERING; FRH, FRQ-interacting RNA helicase; FRQ, Frequency; LHY, LATE ELONGATED HYPOCOTYL; LUX, LUX ARRHYTHMO; Per, Period; PRR, PSEUDO-RESPONSE REGULATOR; PTO, posttranscriptional oscillator; *tim*, timeless; TOC1, TIMING OF CAB EXPRESSION 1; TTFL, transcriptional-translational feedback loop; WC, White Collar. Illustration design assisted by Takka Design.

Circadian rhythms are driven by an internal timing system regulated at the transcriptional level, which gives rise to gene networks that oscillate with a 24-h cycle. Within these networks are clock genes that control the rhythmic transcription of genes involved in physiology and behavior. Interestingly, circadian clock genes were among the first genes identified as controlling behavior. Following studies by Konopka & Benzer (75), who identified the first circadian mutant—period—in fruit flies, a forward-genetic behavioral screen was implemented in mice. Through this screen, the first circadian mutant mouse was identified, followed by the cloning of the first mammalian circadian gene, Clock (70, 159). Research into the mechanisms of mammalian circadian rhythms then exploded, with many additional genes being added to the clock core loop (124, 145). Circadian rhythms are regulated by internal timing systems driven by TTFLs. These feedback loops give rise to gene networks that oscillate in a 24-h cycle, governing critical physiological and behavioral rhythms (Figures 1 and 3). In mammals, key circadian activators such as BMAL1 and CLOCK play central roles in these loops. There are many additional layers of regulation of the mammalian circadian system that we cover below, such as chromatin regulation and posttranslational modifications. One possibility is that similar TTFL mechanisms may operate in parasitic organisms, although they are less understood and potentially adapted to the unique life cycles of these parasites.

### 2.1. Transcriptional-Translational Circadian Regulation in Model Organisms

The molecular components and feedback loops that drive circadian rhythms vary significantly across different kingdoms of life, yet they all achieve a similar goal: maintaining robust, self-sustaining ~24-hour oscillations. By examining the distinct strategies employed by mammals, invertebrates, plants, and fungi, we can appreciate the convergent evolution of timekeeping systems. Each of these model organisms provides a unique perspective on the fundamental principles of circadian biology, revealing a diverse array of molecular components, from transcriptional activators to posttranslational modifiers, that work in concert to regulate daily gene expression and physiology.

#### 2.1.1. Mammals.

In mammals, circadian rhythms are widely controlled by the BMAL1/CLOCK complex. This heterodimer forms the core of the TTFL, where it binds to E-box elements in the promoters of clock-controlled genes (CCGs), activating their transcription (145). The BMAL1/CLOCK complex initiates transcription of Period (*Per*) and Cryptochrome (*Cry*) genes, which, after translation and accumulation, inhibit BMAL1/CLOCK activity (110). RE-VERB and ROR proteins add an additional layer of regulation to *Bmal1* transcription, which is in turn regulated by the rhythmically transcribed Albumin D site-binding protein (DBP), contributing to the three interconnected loops that provide the robustness and adaptability of the circadian system (41, 117, 145). The core clock mechanism is further regulated through post-translational modifications, where Casein Kinase 1 (CK1) phosphorylates PER proteins to control their stability and nuclear translocation, while the F-box protein FBXL3 mediates the proteasomal degradation of CRY proteins through ubiquitination (43). These posttranslational modifications fine-tune the timing of the molecular oscillator by controlling protein stability, activity, and the subcellular localization of clock components. In mammals, approximately 10–15% of genes show circadian oscillations in a tissue-specific manner, with half of the transcriptome being under circadian control in some tissue in the body (99, 169).

The BMAL1/CLOCK complex interacts with the histone acetyltransferases (HATs) p300 and CREB-binding protein (CBP), respectively. Additionally, CLOCK possesses an intrinsic HAT activity that targets H3K9 and H3K14, and together this big transcription complex acetylates histones, which provides an accessible chromatin state for transcription (31, 37). Additionally, the complex recruits other chromatin modifiers such as the methyltransferase MLL1, which deposits activating trimethylation (H3K4me3) marks (67). The rhythmic recruitment of these epigenetic modifiers leads to daily cycles of histone modifications at CCG promoters. Conversely, the PER/CRY repressor complex associates with histone deacetylases and other chromatin remodeling factors that promote a more condensed, transcriptionally repressive chromatin state (34).

The interplay of phosphorylation, protein stability, shuttling between cellular compartments, and auxiliary regulatory feedback loops ensures the precision of the approximately 24-h circadian cycle (43). This self-sustaining system not only maintains a 24-h rhythm but also allows for the modulation of various physiological processes, including metabolism, immune function, and sleep cycles (6). Of note, more than 20 years after the first mammalian clock genes were identified, a novel clock gene was recently reported—RUVBL2, a P-loop NTPase enzyme. RUVBL2 showed a remarkably slow ATPase activity, resembling the well-characterized KaiC-based clock in cyanobacteria, and its disruption led to altered circadian locomotor activity (82). While homologs of BMAL1 and CLOCK have not been identified in malaria parasites or other protozoan parasites, the existence of intrinsic rhythms in these organisms could be through the use of alternative transcription factors or pathways to establish similar feedback mechanisms (124).

#### 2.1.2. Invertebrates.

In *Drosophila*, the core molecular clock mechanism follows a similar TTFL principle as in mammals, but with some key molecular differences (48). The core complex consists of Clock (CLK) and Cycle (CYC; a homolog of mammalian BMAL1), which drive rhythmic transcription through E-box elements. This CLK/CYC heterodimer activates the transcription of period (*per*) and timeless (*tim*) genes, rather than the *Cry* genes found in mammals (1).

A notable distinction in flies is that Cryptochrome (CRY) functions primarily as a blue-light photoreceptor rather than as a transcriptional repressor like its mammalian counterparts (109). Upon exposure to light, CRY undergoes a conformational change that allows it to bind to TIM, triggering TIM's degradation via the proteasome pathway (114). This direct light sensitivity of CRY provides a mechanism for environmental light to reset the circadian clock, making it a key component of light entrainment in insects (151). The system is further regulated by kinases and phosphatases that control the timing of nuclear entry and the stability of clock proteins, ensuring proper periodicity of the circadian cycle (71, 150). In the *Drosophila* brain, approximately 6% of genes show daily oscillations in expression (57). Most of the knowledge generated around the molecular circadian clock mechanism of the fruit fly can be transferred to mosquitos, including malaria's main vector genus *Anopheles* (128, 165). A key interesting difference is that *Anopheles gambiae* possess two CRY proteins: CRY1, which is similar to the photosensitive CRY of the fruit fly, and CRY2, potentially a transcriptional repressor that is similar to the mammalian one (170).

#### 2.1.3. Plants.

Unlike the mammalian BMAL1/CLOCK or insect CLK/CYC systems, plants have evolved a distinct circadian clock architecture that centers on reciprocal regulation between morning- and evening-expressed components. This system utilizes transcription factors that lack direct homologs to the core clock proteins found in animals (132). Circadian clocks in plants regulate a wide range of processes, from growth and development to metabolism and responses to abiotic and biotic stresses.

Plants show particularly extensive circadian regulation, with studies in *Arabidopsis* revealing that 30–40% of expressed genes cycle under constant conditions (26). The morning-expressed single-myeloblastosis (MYB) domain transcription factors CCA1 (CIRCADIAN CLOCK ASSOCIATED 1) and LHY (LATE ELONGATED HYPOCOTYL) function as transcriptional repressors, directly binding to and inhibiting the expression of evening genes. These are temporally followed by the sequential expression of members of the *PSEUDO-RESPONSE REGULATOR* (*PRR*) gene family (*PRR9*, *PRR7*, *PRR5*, and *PRR1/TOC1*) from early morning to dusk, which in turn act as repressors of CCA1 and LHY. Other genes such as *ELF3* (*EARLY FLOWERING 3*), *ELF4*, and *LUX* (LUX ARRHYTHMO) are expressed at night and form an evening complex that represses gene expression, ultimately contributing to the reactivation of CCA1 and LHY expression (101, 104).

#### 2.1.4. Fungus.

The fungal circadian system, while following the basic principle of a TTFL like mammals and insects, employs distinct molecular components. Unlike the mammalian BMAL1/CLOCK, insect CLK/CYC, or plant CCA1/LHY-PRR systems, fungi use the White Collar complex (WCC) as the primary transcriptional activator, and instead of PER/CRY, PER/TIM, or PRR proteins, they employ the Frequency (FRQ)/FRQ-interacting RNA helicase (FRH) complex as the negative element (4, 63).

At the core of the oscillatory system, two transcription factors, White Collar-1 (WC-1) and White Collar-2 (WC-2), form the WCC and drive gene expression by binding to the promoter region of the frequency (*frq*) gene (42). This leads to the production of the FRQ protein, which partners with the FRH to form the negative arm of the feedback loop (23). Once translated, this FRQ/FRH complex enters the nucleus and promotes the phosphorylation of the WC transcription factors, thereby inhibiting their activity (50). As the day progresses, FRQ itself becomes progressively phosphorylated by multiple kinases [e.g., Casein Kinase-1a (CK-1a) and Casein Kinase-2 (CK-2)], leading to its degradation and allowing the cycle to begin anew (149).

Through this oscillatory mechanism, the WCC regulates 10–40% of the transcriptome through both direct and indirect means (58, 59). Direct regulation occurs through WCC binding to promoter regions of first-order CCGs, while indirect control is achieved through the rhythmic activation of downstream transcription factors that regulate second-order CCGs (140). These CCGs orchestrate crucial cellular processes including metabolism, protein synthesis and secretion, cell signaling, and stress responses. Their temporal separation helps the organism anticipate and adapt to daily environmental changes while optimizing resource utilization (58).

The circadian regulation extends beyond transcriptional control, forming an intricate network of regulation at multiple levels. While approximately 40% of the transcriptome shows circadian oscillations, there is not always a direct correlation between rhythmic transcripts and rhythmic proteins, indicating extensive posttranscriptional and posttranslational regulation (25, 59). Similar discordance between rhythmic transcripts and proteins has been observed in mammals, where only ~5% of rhythmic proteins correspond to rhythmic transcripts in the liver (89, 126). In both systems, the clock achieves this complex coordination through the modulation of messenger RNA (mRNA) splicing, antisense transcription, RNA stability, translation, and protein activity or degradation (73, 131).

#### 2.1.5. Bacteria.

Unlike the TTFLs that characterize eukaryotic circadian systems (mammals, insects, plants, and fungi), the cyanobacterial circadian clock is primarily centered on post-translational modifications. The best understood bacterial clock is that of the cyanobacterium *Synechococcus elongatus*, which centers on the KaiABC protein complex (60). The molecular mechanisms of this unique timekeeping system will be further explored in Section 3.1.

### 2.2. Transcriptional Regulation of *Plasmodium*

*Plasmodium* life cycle progression is accompanied by important transcriptional changes at both population and single-cell levels, across different stages within human erythrocytes and hepatocytes, and in mosquitoes (13, 53, 55, 78, 80, 84, 108, 121, 130, 154, 160). To ensure this dynamic gene expression is properly coordinated with environmental factors and developmental stages, *Plasmodium falciparum* employs conserved eukaryotic transcriptional machinery components, including the preinitiation complex (PIC), mediator complex, TATA-binding proteins, and the Spt-Ada-Gcn5 acetyltransferase (SAGA) complex. The genome also encodes putative sequence-specific transcription factors, including 13 homeodomain-like (HD) proteins, 10 MYB proteins, 3 high mobility group box (HMGB) proteins, and around 170 zinc finger domain proteins (reviewed in 153).The largest and best characterized family of transcription factors in *P. falciparum* is the apicomplexan AP2 (ApiAP2) family with approximately 30 members.

#### 2.2.1. AP2-transcription factors in development and stress response.

ApiAP2 transcription factors regulate gene expression throughout the complex *Plasmodium* life cycle, controlling developmental transitions and responses to environmental changes across both vertebrate and mosquito stages (reviewed in 139). In sexual development regulation, PfAP2-G acts as the master regulator of sexual commitment and gametocytogenesis (66), while PfAP2-G2 and PfAP2-G5 function as transcriptional repressors regulating gametocyte development (136, 137, 166). PfAP2-FG/G3 is critical for female gametocyte development (168), and PfAP2-Z controls zygote development (102).

In asexual development and stress response, PfAP2-P mediates the activation of genes involved in host cell remodeling, parasite development, and invasion (142), while PfAP2-I specifically regulates merozoite invasion-related genes (133). PfAP2-EXP regulates host cell–exported multigene families (e.g., *rif*, *stevor*) during the asexual intraerythrocytic cycle (88). PfAP2-Sp regulates sporozoite gene programs and is required for sporozoite development (167). PfAP2-O2 controls ookinete development and early blood stages (96, 137), and PfAP2-HS regulates heat-shock responses in blood stages (152).

These proteins contain one to three AP2 DNA-binding domains that recognize a wide variety of DNA motifs. Unlike plant AP2/ERF transcription factors that all recognize a GCC-box motif, ApiAP2 transcription factors in *P. falciparum* recognize over 20 unique DNA motifs (18). This DNA binding is context-dependent, influenced by chromatin structure, accessibility, and DNA modifications. Interestingly, some transcription factors that preferentially bind divergent DNA motifs may bind overlapping genomic regions due to low-affinity binding to other sequence motifs (11).

#### 2.2.2. Regulatory mechanisms and networks.

ApiAP2 proteins employ sophisticated regulatory mechanisms, including autoregulation and feedforward loops. Autoregulation, where transcription factors regulate their own expression, is common among ApiAP2 proteins (137). This self-regulation mechanism potentially reduces biosynthetic costs and allows rapid response to environmental cues, thereby maintaining stable gene expression patterns (2).

The network features numerous feedforward loops that provide precise temporal control of gene activation and repression. PfAP2-G exemplifies this through a positive feedforward circuit regulating sexual differentiation. Once activated, PfAP2-G enhances its own expression while simultaneously activating downstream sexual development genes (65, 66). Similarly, PfAP2-G5 and PfAP2-G2 form a regulatory loop controlling gametocytogenesis, where they exert opposing effects on early gametocyte gene expression (136, 137, 166). PfAP2-HC participates in another feedforward system regulating var genes, which is crucial for antigenic variation and immune evasion (20, 137).

These regulatory mechanisms are enhanced by extensive cross talk between ApiAP2 factors. Key regulators like PfAP2-G5, PfAP2-G2, and PfAP2-HC control both their direct target genes and other ApiAP2 transcription factors. Some ApiAP2 proteins show cooperative regulation, such as PfAP2-P, PfAP2-I, and PfAP2-G coordinating genes involved in both erythrocyte invasion and sexual commitment (142). This complex interplay enables precise control of gene expression throughout the parasite's development cycle (137).

#### 2.2.3. Beyond AP2 transcription factors: expanding the network of DNA-binding proteins.

While ApiAP2 transcription factors have been the primary focus of transcriptional regulation studies in *Plasmodium* over the last two decades (5), the parasite possesses a rich array of other DNA-binding proteins. The genome encodes 196 DNA-binding proteins, including 13 HD proteins, 10 MYB family proteins, 3 HMGB domain proteins, and 170 zinc finger domain proteins (10). Novel studies have begun to reveal how these non-AP2 proteins contribute to parasite development and differentiation.

A pioneering discovery in this field was the characterization of homeodomain protein 1 (HDP1), the first transcription factor among the 13 homeodomain proteins to be functionally described in *Plasmodium* (19). HDP1 contains a C-terminal HD DNA-binding domain and is specifically expressed during sexual development. As a nuclear protein, HDP1 shows remarkable chromatin association, with about 70% of the protein remaining bound even under high salt conditions (19). HDP1 functions through sequence-specific DNA binding to GC-rich motifs (GTGCAC) and recruitment of chromatin remodeling machinery (19).

Chromatin immunoprecipitation sequencing (ChIP-seq) analysis revealed HDP1's involvement in regulatory feedback loops through binding upstream of its own locus (19, 103). Furthermore, HDP1 regulates two ApiAP2 genes essential for later stages of sexual development, including AP2-O2, demonstrating cross-regulation between different classes of transcription factors (19, 103).

These discoveries challenge the AP2-centric view of transcriptional regulation in *Plasmodium* and suggest equally important roles for other DNA-binding proteins (19). The characterization of the remaining homeodomain proteins and other DNA-binding factors may reveal additional regulatory networks controlling various aspects of the parasite life cycle, including the regulation of the clock.

#### 2.2.4. Chromatin-based regulation.

In *P. falciparum*, like other eukaryotes, genomic DNA is organized into chromatin through its association with nucleosomes, each composed of a histone octamer containing two units of H2A, H2B, H3, and H4. This chromatin structure plays a crucial role in transcriptional regulation through both nucleosome positioning and histone modifications. Unlike most eukaryotes, apicomplexan parasites and Microsporidia lack linker histone H1, which in the case of *P. falciparum* partially explains the lack of higher-order compaction of nuclear DNA (28, 144). Nonetheless, similar to other eukaryotes, active promoters typically display nucleosome-depleted regions upstream of transcription start sites, allowing binding of the preinitiation complex (PIC), while silenced genes show higher nucleosome occupancy that prevents PIC interaction (17, 68, 116).

The parasite employs various chromatin remodeling complexes that work in concert with transcription factors to regulate gene expression. ApiAP2 transcription factors interact with these complexes across different chromatin states—PfAP2-LT partners with the PfSAGA complex in euchromatic regions (94), while PfAP2-HC operates in heterochromatic regions through interactions with PfHP1 (14, 20, 137).

A key chromatin remodeling complex is PfMORC (microrchidia), built around a single MORC protein containing an N-terminal ATPase domain and C-terminal coiled-coil domains for protein-protein interactions (24). A recent study has revealed that PfMORC is essential for parasite survival and plays a crucial role in modulating chromatin structure and heterochromatin formation throughout the erythrocytic cycle (22). PfMORC orchestrates chromatin organization by partnering with multiple ApiAP2 proteins, including PfAP2-P, PfAP2-G5, and PfSIP2 (22, 138). Through its association with the PfISWI chromatin remodeling complex, PfMORC regulates subtelomeric var genes and mediates chromosomal interactions (16, 138).

ChIP-seq and Hi-C studies have demonstrated PfMORC's crucial role in maintaining heterochromatin structure and regulating stage transitions (22). Its downregulation impairs key histone marks and leads to heterochromatin collapse, resulting in parasite death (22). At euchromatic boundaries, PfMORC collaborates with PfAP2-P and PfAP2-I to regulate genes involved in red blood cell egress and invasion (142).

Recently, PbARID (an AT-rich interactive domain-containing protein) has emerged as another crucial chromatin regulator. Its ARID domain, a helix-turn-helix motif-based DNA-binding domain conserved across eukaryotes, shows the closest relation to the ARID2 subfamily of the SWI/SNF chromatin remodeling complex (103). PbARID exhibits dual functionality in chromatin remodeling during gametocyte development through distinct partnerships. The transcription factor HDP1 recruits PbARID to TGCACA motifs to regulate genes important for both male and female gametocyte development. Additionally, PbARID partners with gSNF2, the ATPase subunit of the SWI/SNF complex, to bind TGTCT motifs and activate male gametocyte-specific genes. RIME (rapid immunoprecipitation mass spectrometry) analysis identified seven putative SWI/SNF complex subunits, with six conserved between yeasts and humans (103).

The essential nature of PbARID is demonstrated by its role in gametocyte development—its disruption leads to complete loss of male gametocytes and abnormal female gametocyte development. ChIP-seq analysis revealed that PbARID's targets include both male- and female-enriched genes, with significant downregulation in PbARID-knockout parasites, indicating its function as a transcriptional activator through chromatin remodeling (103). This sophisticated regulatory mechanism exemplifies how *Plasmodium* achieves complex developmental control despite its limited transcription factor repertoire (5, 103).

This sophisticated interplay between different families of *Plasmodium* transcription factors and chromatin-associated proteins creates a flexible regulatory system that enables precise control of developmental processes and appropriate responses to environmental conditions.

## 3. POSTTRANSCRIPTIONAL FEEDBACK LOOPS

While transcriptional regulation through TTFL mechanisms is prevalent in eukaryotes, some organisms have evolved alternative timing systems based primarily on posttranslational modifications. Although in most cases it will lead to rhythmic transcriptional regulation, these systems demonstrate that circadian rhythms can be generated and maintained through different molecular architectures.

### 3.1. Cyanobacteria and Other Bacterial Clocks

The cyanobacterial KaiABC system represents the best understood prokaryotic circadian clock. In *S. elongatus*, three proteins—KaiA, KaiB, and KaiC—form an oscillator that generates 24-h rhythms through oscillating phosphorylation states of KaiC, regulated by KaiA and KaiB (60, 100, 129). The remarkable feature of this system is its ability to maintain rhythmicity even when reconstituted in vitro with just these three proteins and ATP, demonstrating that transcription is not essential for circadian timing (100, 155).

Environmental synchronization of the cyanobacterial clock occurs through metabolic entrainment rather than direct light sensing, primarily through sensitivity to cellular ATP/ADP ratios that align the clock with peak photosynthetic activity (reviewed in 111). The system also responds to nighttime-associated photosynthetic metabolites, providing additional temporal cues (62, 69).

The Kai phosphorylation oscillator controls gene expression by modulating the phosphorylation state of RpaA, a transcription factor (87, 147). Phosphorylated RpaA activates dusk genes and indirectly represses dawn genes, creating a TTFL that stabilizes the clock phase (47, 87). This system demonstrates how posttranslational modifications can drive circadian rhythms while still integrating with transcriptional control mechanisms (Figure 3).

Recent research has revealed that KaiB and KaiC homologs exist beyond cyanobacteria, appearing in other bacterial species and archaea (135). These homologs serve diverse functions across different species. In *Legionella pneumophila*, they regulate stress responses (85), while in *Rhodopseudomonas palustris*, they are considered an ancestral system (primordial clocks) that is based on KaiC and KaiB proteins and includes some, but not necessarily all, of the canonical properties of circadian clocks (86). An interesting variation appears in *Rhodobacter sphaeroides*, where the system functions as a timer without requiring KaiA (115).

Beyond cyanobacteria, several bacteria display circadian-like behaviors through yet unknown regulatory mechanisms. For instance, the soil bacterium *Bacillus subtilis* exhibits temperature-compensated 24-h rhythms that can be entrained by both light and temperature cycles, though the underlying molecular mechanism remains unclear (36, 134). Similarly, the gut bacterium *Klebsiella aerogenes* shows temperature-entrainable circadian rhythms in swarming motility (112, 113). These findings suggest that circadian timing mechanisms may be more widespread among bacteria than previously recognized, with different species potentially employing TTFL, PTFL, or hybrid systems adapted to their specific ecological niches, though they likely evolved multiple times using different molecular components.

The evolutionary connection between cyanobacterial circadian systems and other organisms is particularly intriguing in the case of apicomplexan parasites like *Plasmodium*, which contain a vestigial plastid called the apicoplast (90). This organelle originated through secondary endosymbiosis of a photosynthetic unicellular alga, though the precise nature of this ancestral alga remains debated (158). The apicoplast retains multiple essential metabolic functions, including the synthesis of fatty acids, isoprenoids, and iron-sulfur clusters (83, 91, 119), despite having lost its photosynthetic capability during evolution to an intracellular parasitic lifestyle (64).

Some proteins of cyanobacterial origin have been retained and repurposed for apicoplast function, as demonstrated by AMR4, a CaaX-like protease required for apicoplast biogenesis (92), and several other proteins involved in organelle maintenance and metabolic pathways (12, 157). Recently, a nuclear-encoded σ70-like sigma factor (ApSigma) was identified in *P. falciparum* that regulates apicoplast gene expression and responds to host hormones (72). This sigma factor, similar to those found in bacteria and plant chloroplasts, contains conserved domains for promoter recognition and RNA polymerase binding. This could be a mechanism by which parasites might synchronize apicoplast function with host circadian rhythms.

### 3.2. Peroxiredoxins and Metabolic Rhythms

Peroxiredoxins (PRXs) are highly conserved antioxidant proteins that undergo ~24-h cycles of oxidation reduction across many organisms (35). In human red blood cells, which lack nuclei and therefore cannot perform transcription, PRX proteins display circadian rhythms through the reversible oxidation of their catalytic cysteine residues (107). This oxidation is thought to be driven by hydrogen peroxide generated through hemoglobin auto-oxidation and is countered by NADPH-dependent reduction pathways (35).

The rhythmic oxidation of PRX is tightly coupled with glucose metabolism through both glycolysis and the pentose phosphate pathway (PPP) (21). These pathways show opposite phases throughout the circadian cycle—PPP flux peaks during the subjective day aligned with maximum PRX oxidation, while glycolytic activity is highest during the night. Inhibition of either pathway abolishes both metabolic oscillations and PRX redox rhythms (21).

In *Plasmodium* parasites, PRXs are particularly important as they lack other major antioxidant enzymes like catalase and glutathione peroxidase (44). It has been shown that parasites can uptake PRX during the blood-stage infection (74). During development in the mosquito blood meal, *Plasmodium* ookinetes strongly upregulate cytosolic PRXs in response to oxidative challenges (156). Beyond their antioxidant role, *P. falciparum* PRX (Prx1a) interacts with over 127 cytosolic proteins involved in essential processes, including carbohydrate metabolism and signal transduction, significantly impacting target enzyme activities (15). Given these regulatory functions and the rhythmic oxidation patterns seen across organisms, PRXs may help coordinate the parasite's metabolic adaptation to daily redox cycles throughout its complex life cycle.

### 3.3. Noncoding RNAs

Posttranscriptional regulation is a key layer of the regulation of circadian systems, yet their mechanisms are less well-defined (46). Recent advances in genomic technologies have revealed that a large portion of eukaryotic genomes is transcribed into ncRNAs. In mammals, up to 80% of the genome is actively transcribed, yet only about 2% encodes proteins (30, 32). Among ncRNAs, long noncoding RNAs (lncRNAs) are defined as transcripts longer than 200 nucleotides that do not encode proteins. Despite their low expression levels and limited sequence conservation across species, lncRNAs play crucial roles in various biological processes, including circadian rhythm regulation (76).

In mammals, lncRNAs display distinct spatial and temporal expression patterns, with only a small fraction being ubiquitously expressed (29). Like protein-coding genes, 5–40% of lncRNAs show circadian oscillation in their expression, varying by tissue type (169). These rhythmic lncRNAs are regulated by core clock transcription factors, including BMAL1/CLOCK, which bind to E-box elements in their promoter regions (73). For instance, the noncoding repressor of NFAT (NRON) has been shown to regulate the circadian clock by interacting with the PER2 protein at the perinuclear domain, inhibiting its phosphorylation and nuclear translocation (79). The knockdown of NRON leads to longer circadian periods and reduced amplitude, demonstrating its importance in clock regulation (79).

Several other lncRNAs have been identified as important regulators of circadian genes. Lnc-Crot, a nuclear lncRNA rhythmically expressed in liver and kidney, forms chromatin loops and serves as a platform for coordinating the rhythmic expression of distant genes (38). In liver cancer, HULC lncRNA stabilizes CLOCK mRNA by binding directly to it, while UCA1 lncRNA protects CLOCK from repression by blocking microRNA miR-206 (27, 56). In the pineal gland, another lncRNA, TCONS_00044595, helps maintain CLOCK levels during low-oxygen conditions (81). These examples highlight the diverse mechanisms through which lncRNAs can influence circadian gene expression, including chromatin looping, mRNA stability, microRNA sponging, and transcriptional regulation.

The function of ncRNAs in circadian biology appears to be evolutionarily conserved across different species. In *Neurospora crassa*, the antisense transcript of the frequency gene (qrf) shows rhythmic expression antiphasic to frq and regulates light-induced phase shifts (162). Similarly, in *Drosophila*, temperature-dependent splicing of the period gene regulates an antisense transcript called Daywake (dyw), which controls daytime sleep behavior (164). In mammals, the *Per2* gene has an antisense transcript (*Per2AS*) that is expressed rhythmically and antiphasic to *Per2* mRNA in mouse liver (73, 93). *Per2AS* forms a feedback loop with *Per2*. This and possibly other interactions with clock proteins lead to *Per2AS* affecting the amplitude of circadian rhythms (97).

In *P. falciparum*, recent studies have identified over 1,700 lncRNAs, with many showing stage-specific expression patterns throughout the parasite's life cycle (7). Similar to mammalian lncRNAs, these transcripts can be categorized based on their subcellular localization, with about 41% enriched in the nuclear fraction and 11% in the cytoplasmic fraction. While the direct role of these lncRNAs in circadian regulation remains to be established, several have been implicated in critical developmental processes, including sexual differentiation and gametocyte development. For instance, *gdv1-as*-lncRNA and *md1*-lncRNA regulate sexual commitment and sex determination, respectively (40, 45), highlighting the importance of lncRNAs in regulating key stages of the parasite's life cycle.

## 4. CONCLUSION

The diverse mechanisms controlling biological timing across the tree of life reveal both conserved principles and lineage-specific innovations. From the well-characterized TTFL systems in mammals and insects to the elegant posttranslational oscillator of cyanobacteria, organisms have evolved multiple solutions to the challenge of maintaining 24-h rhythms (8, 145). This review highlights how parasites like *Plasmodium* may represent yet another variation on biological timing, combining elements of both transcriptional and posttranslational regulation.

The discovery of intrinsic rhythms in *Plasmodium* raises fundamental questions about their timing mechanisms. While these parasites lack canonical clock genes, they possess sophisticated regulatory machinery, including ApiAP2 proteins, homeodomain transcription factor, and other DNA-binding proteins (10, 19, 139), along with extensive chromatin-based regulation (52, 138) and ncRNA networks (7, 163). These components could form novel timing circuits distinct from known circadian mechanisms.

The evolutionary history of the apicoplast suggests possible connections to bacterial timing systems (91), while host-parasite interactions may have driven the development of unique synchronization mechanisms. Future research should focus on identifying the molecular components of parasite timing systems and understanding their integration with host rhythms, potentially revealing new therapeutic targets. How can we identify the clock genes of *Plasmodium*? One possibility is forward genetic screens, as was successfully done for flies, mice, and *Neurospora* (39, 75, 146, 159). Alternatively, DNA baits—an approach that has worked in plants—could help uncover key regulators (161). The rapid advancements in bioinformatics and the omics era may also offer new strategies for identifying the clock. Ultimately, only time will tell. As new technologies enable deeper investigation of parasite biology at multiple scales, these discoveries could both advance our understanding of parasite biology and reveal new paradigms in biological timing.
